# Draft genome of *Gongronella butleri* reveals the genes contributing to its biodegradation potential

**DOI:** 10.1186/s43141-022-00351-2

**Published:** 2022-05-18

**Authors:** Ravisankar Valsalan, Deepu Mathew, Girija Devaki

**Affiliations:** 1 Bioinformatics Centre, Kerala Agricultural University, Thrissur, 680656 India; 2 Department of Agricultural Microbiology, College of Agriculture, Kerala Agricultural University, Thrissur, 680656 India

**Keywords:** Catalysis mechanisms, Composting pathway, Genome annotation, NGS, Solid biowaste

## Abstract

**Background:**

*Gongronella butleri* is a fungus with many industrial applications including the composting of solid biowaste. Kerala Agricultural University, India, has developed a microbial consortium of which GbKAU strain of *G. butleri* is a major component. Even with great industrial significance, genome of this fungus is not published, and the genes and pathways contributing to the applications are not understood. This study had the objective to demonstrate the solid biowaste decomposing capability of the strain, to sequence and annotate the genome, and to reveal the genes and pathways contributing to its biodegradation potential.

**Results:**

Strain GbKAU of *G. butleri* isolated and purified from the organic compost was found to produce higher levels of laccase and amylase, compared to *Bacillus subtilis* which is being widely used in biosolid waste management. Both were shown to be equally efficient in the in vivo composting capabilities. Whole genome sequencing has given ~11 million paired-end good quality reads. De novo assembly using dual-fold approach has yielded 44,639 scaffolds with draft genome size of 29.8 Mb. A total of 11,428 genes were predicted and classified into 359 groups involved in diverse pathways, of which 14 belonged to the enzymes involved in the degradation of macromolecules. Seven previously sequenced strains of the fungus were assembled and annotated. A direct comparison showed that the number of genes present in those strains was comparable to our strain, while all the important biodegrading genes were conserved across the genomes. Gene Ontology analysis had classified the genes according to their molecular function, biological process, and cellular component. A total of 104,718 SSRs were mined and classified to mono- to hexa-nucleotide repeats. The variant analysis in comparison with the closely related genus *Cunninghamella* has revealed 1156 variants.

**Conclusions:**

Apart from demonstrating the biodegradation capabilities of the GbKAU strain of *G. butleri*, the genome of this industrially important fungus was sequenced, de novo assembled, and annotated. GO analysis has classified the genes based on their functions, and the genes involved in biodegradation were revealed. Biodegradation potential, genome features in comparison with other strains, and the functions of the identified genes are discussed.

**Supplementary Information:**

The online version contains supplementary material available at 10.1186/s43141-022-00351-2.

## Background

Disposal of solid biowaste has always been quite challenging, and the composting processes have reasonably addressed this issue. Fungi such as *Trichoderma viride*, *Trichoderma reesei*, *Candida rugopelliculosa*, *Aspergillus niger*, *Aspergillus flavus*, *Aspergillus oryzae*, *Chaetomium thermophilum*, *Sclerotium* and white rot fungi *Fomes fomentarius*, *Phanerochaete chrysosporium*, and *Trametes versicolor* and bacteria such as many species of *Bacillus* especially *B. casei*, *B. thermoamylovorans*, *B. brevis*, *B. coagulans*, *B. licheniformis*, *B. subtilis*, *B. tequilensis*, *B. venezuelans* and *B. amyloliquefaciens*, *Lactobacillus buchneri*, *Pediococcus acidilactici*, *Pseudomonas aeruginosa*, *Streptomyces* sp., *Micromonospora* sp., and *Cellulomonas* sp. are efficient for solid biowaste composing through extracellular secretion of Cellulase and Laccase [1–4]. Additionally, thermotolerant lipolytic actinomycete, *Thermoactinomyces vulgaris*, and yeast *Pichia kudriavzevii* were also proven effective [5, 6]. The Western Ghats of India has been a source of many of the industrially important microbes, especially those yielding biodegradative enzymes.

The *Gongronella* spp. are among the most commonly occurring and economically important members of the Zygomycetes class which produces chitosan, an important component of the cell wall of these fungi [7, 8]. *Gongronella butleri* (Lendn.) Peyronel & Dal Vesco, a key member of the species, is one of the most important organism used for commercial chitosan production. Chitosan has received worldwide attention as a promising renewable polymeric material with extensive applications in industry and agriculture, such as cholesterol absorption, semipermeable membrane production, antifungal agent, plant growth elicitor, and heavy metal chelator [9–12]. The species has been described to have biocomposting potential in combination with other organisms such as *Bacillus subtilis*, *Bacillus niabensis*, and *Meyerozyma guilliermondii* [13, 14]. *G. butleri* is reported to have the highest cellulase and protease activities among the microorganism studied for the biocomposting ability. This fungus is also useful in the biotransformation of 3-keto-androstanes. Its capability to effectively carry out the hydroxylation of 3-keto-4-ene-androstane steroids is industrially significant since the intermediates are useful in pharmaceutical preparations [15]. The species also produces β-glucosidases, which have applications in industrial processes such as biofuel production, winemaking, and development of functional foods [16]. They have also been used in the stereoselective reduction of ketones [17] and possess the potential for quicker amylase production in low-cost culture media such as agro-industrial residues [18]. In addition, eight new 2-pentenedioic acid derivatives were isolated from this Zygomycetes fungus [19]. A recent study has also revealed its involvement in the root rot disease of mulberry [20]. Although previous studies report the wide and important biotechnological application of this fungus, little efforts have been made on its genomic analysis.

This study reports the isolation and purification of *Gongronella butleri*, assessment of its biodegradative enzyme production and in vivo bio-solid waste composting potential, and the first draft genome of this fungus based on sequencing on Illumina HiSeq-2500 platform followed by de novo assembly. Gene prediction and annotation along with downstream analysis have identified the gene models, with special reference to biodegradation. Comparative genome analysis with the previously sequenced strains of this fungus was also carried out.

## Methods

### Fungus isolation and morphological confirmation

The *G. butleri* strain GbKAU was isolated and purified from the organic compost at Kerala Agricultural University, India, following the standard protocol [21]. Cultural characteristics such as colony appearance and mycelial texture were observed by growing the culture on potato dextrose agar medium (PDA) and incubating at 28 °C for 3–7 days. Pure isolates were obtained by picking fungal tips. Microscopic analysis was carried out by staining with lactophenol cotton blue stain. Observations were made using a camera-supported microscope (Leica ICC50, Germany) at 40× magnification. The type of mycelium, characteristics of sporangia, and arrangement of sporangiophore were recorded. The identity was cross-verified in the studies conducted at the National Center of Fungal Taxonomy, New Delhi.

### Assay of degradative enzyme production by G. butleri

Production of the enzymes involved in the degradation of cellulose (cellulase C1 + Cx and glucanase), starch (amylase), and lignin (laccase) by *G. butleri* was assayed in comparison with *Bacillus subtilis*. In our previous studies, *B. subtilis* was shown as the second best microbe for the composting [13], and this microbe is also reported to be efficient by many researchers in biosolid waste management [22, 23]. Hence, for the comparative evaluation of the biodegradation potential of *G. butleri* strain, *B. subtilis* was used.

### Glucanase assay

Cellulase activity was determined using DNS (3,5-dinitrosalicylic acid) method. The fungal isolate was grown in CMC broth for 72 h at 37 ± 2 °C and centrifuged at 10,000 rpm for 10 min. A total of 0.5 mL of the supernatant enzyme solution was mixed with 0.1 M citrate buffer (pH 5.0) containing 1.0% cellulose substrate. The resulting reaction mixture was incubated at 55 °C for 15 min. After reaction, 3.0 mL of DNS reagent was added, and this mixture was boiled for exactly 5 min to terminate the reaction in a vigorously boiling water bath, and 1.0 mL of potassium sodium tartrate solution was added. After cooling the tubes to room temperature, absorbance was recorded by spectrophotometer at 540 nm against the blank without enzyme filtrate. The reducing sugar concentrations were calculated using glucose standard curve.

### Filter paper assay for total cellulase activity

Culture supernatant (0.5 mL) was transferred to a clean test tube, and 1 mL of 0.1 M sodium citrate buffer (pH 5.8) was added. A Whatman no. 1 filter paper strip (6 cm × 1 cm) of 32 mg was then added to each tube. Tubes were incubated in a water bath at 50 °C for 1 h, and 3 mL of DNS reagent was added. The tubes were placed in a boiling water bath for 5 min, and the reactions were stopped by addition of 1 mL of 40% sodium potassium tartrate to each tube. After cooling the tubes to room temperature, absorbance was measured at 540 nm. Reducing sugar levels was determined using the DNS method with D-glucose as a standard at 540 nm [24].

### Laccase assay

The laccase activity was assayed at room temperature by using 10 mM guaiacol in 100 mM sodium acetate buffer (pH 5.0). The culture was grown in broth for 3–5 days and the filtrate used as enzyme source. The reaction mixture contained 3.0 mL acetate buffer, 1.0 mL guaiacol, and 1.0 mL enzyme source. The change in the absorbance of the reaction mixture containing guaiacol was monitored at 470 nm for 10 min of incubation using UV spectrophotometer. Enzyme activity was measured in U/ml, which is defined as the amount of enzyme catalyzing the production of one micromole of colored product per min per mL [25].

$$\mathrm{Volume}\kern0.5em {\mathrm{activity}}\kern0.5em \left({\mathrm{U}}/{\mathrm{mL}}\right)=\frac{\Delta {\mathrm{A}}470{\mathrm{nm}}/\min \kern0.5em \times \kern0.5em 4\kern0.5em \times \kern0.5em {\mathrm{Vt}}\kern0.5em \times \kern0.5em {\mathrm{dilution}}\ {\mathrm{factor}}}{\EUR \kern0.5em \times \kern0.5em {\mathrm{Vs}}}$$

where, *Vt* = final volume of reaction mixture (ml) = 5.0, *Vs* = sample volume (mL) = 1.0, *€* = extinction coefficient of guaiacol = 6740/M/cm, and 4 - derived from unit definition and principle.

### Amylase assay

Amylase activity was assayed by DNS method using 1.0% starch as substrate. The reaction mixture containing 1.0 mL of soluble starch solution was mixed with 1.0 mL of the crude enzyme sources and incubated for 15 min at room temperature. After incubation, 2.0 mL of the DNS reagent was added, and the reaction was terminated by immersing the tube in a boiling water for 10 min followed by addition of 1.0 mL of potassium sodium tartrate solution. After cooling, the volume was made up to 10 mL, and the absorbance was read at 560 nm. The reducing sugar concentration was calculated according to glucose standard curve. The enzyme activity was expressed as mg of glucose equivalent released per mL under standard assay conditions.

### In vivo evaluation of G. butleri for bio-solid waste management

The efficiency of *G. butleri* was evaluated in aerobic composting using Thumburmuzhi model [26]. *B. subtilis*, which was earlier reported to perform well in aerobic composting [27], and composting using cow dung were also evaluated for comparison. Biosolid waste and dry leaves were alternately layered in 6 inch thickness, and 250 mL of the inoculum was diluted in 1:4 ratio and sprinkled over the dry leaves. In case of cow dung-based composting, 6-inch layer of cow dung was added after every layer of dry leaves. The filled units were left undisturbed for 80 days, and temperature and height of the pile were measured in every 10 days.

### DNA extraction

For DNA extraction, pure culture was isolated from the conical flask, and protocol by Dellaporta [28] was followed. The fungus was frozen with liquid nitrogen in a mortar, ground to fine powder, and transferred to the tube containing extraction buffer (100 mM Tris pH 8.0, 50 mM EDTA pH 8.0, 500 mM NaCl, 10 mM mercaptoethanol, and 1.25% SDS). After mixing, 5 M potassium acetate was added and incubated at 0 °C for 20 min. Supernatant obtained after centrifugation was poured into a clean tube where genomic DNA was allowed to precipitate with isopropanol for 30 min at −20 °C. After centrifugation, pellet was resuspended in 50 mM Tris, 10 mM EDTA, and pH 8.0 and transferred to a 20 μL tube where DNA was precipitated with 80% ethanol, and the dried pellet was subsequently dissolved in 10 mM Tris, 1 mM EDTA, and pH 8.0.

### Sequencing and assembly

Sequencing of genomic DNA was performed on Illumina HiSeq-2500 platform at 50× coverage. The sequencing quality was assessed using FastQC software [29]. In preprocessing, adapter sequences trimmed, bases above Q30 were considered, and the rest filtered off. A total of ~11 million paired-end good quality reads were retained after preprocessing. These reads were assembled using SPAdes assembler [30], employing different k-mer lengths (21, 33, 55, 77, 99, and 111), setting the --cov cutoff parameter to auto and using the --careful option. For further confirmation of the results from SPAdes, a reassembly was done using ABySS de novo assembly program [31], employing k-mer lengths 32–128 to optimize the assembly. Final output was best at k-mer value of 120.

### Gene prediction, annotation, and variant detection

Gene prediction from the scaffolds and annotation of the genes were performed using the following procedure. Assembly was trained using the training mode in Augustus tool [32] which created a new dataset for this fungus since it was not available in its database. Following a generalized Hidden Markov model, the training dataset created was used to predict the genes in *Gongronella butleri*. Subsequently, the genes predicted were allotted to different pathways using KAAS (KEGG Automatic Annotation Server) database [33]. BBH (b-directional best hit) method, most suited for annotating the complete genomes, was employed for the pathway analysis. For whole genome annotation, predicted genes were further analyzed using BLASTX with default parameters against UniProt protein database [34]. Gene Ontology (GO) was also performed on the predicted genes to identify their functions. Additionally, simple sequence repeats (SSRs) were identified using the predicted genes. To further identify the variants specific to the assembled genome, the sample reads were aligned with *Cunninghamella* species to obtain SNP details. Alignment files were used as input to identify the variants using samtools and bcftools1.9. The significant variants were filtered and identified at cutoff depth of 20 and quality 30.

### Comparative genome analysis with other strains

To compare our sequence with available sequences of *G. butleri*, raw data of seven strains have been downloaded from SRA database, de novo assembled, genes predicted, and the genomes were annotated. No assembled sequences were available in public databases. All analyses were performed following the same procedure that was employed with our strain.

## Results

### Fungus isolation and morphological confirmation

**Fig. 1:**
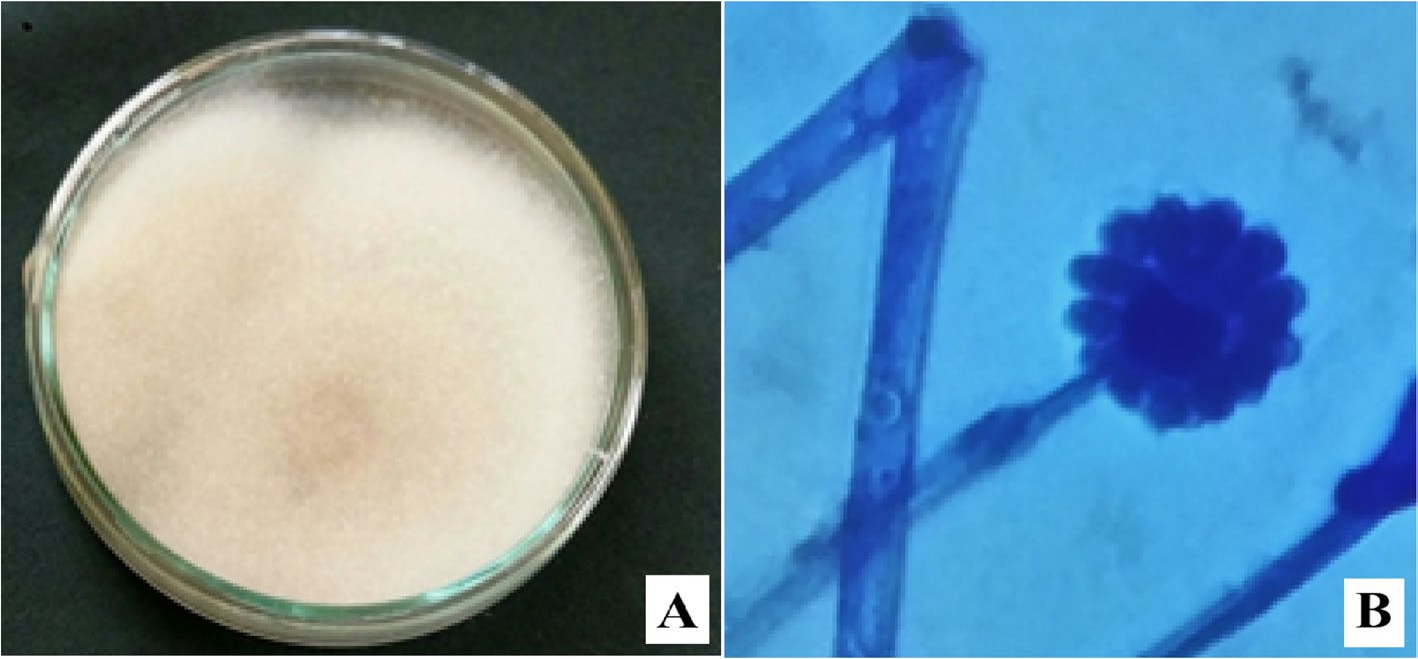
Morphological characteristics of *G. butleri*. **A** Mycelial proliferation on PDA medium. **B** Hyphae and sporangia (under 40× objective)

**Fig. 2:**
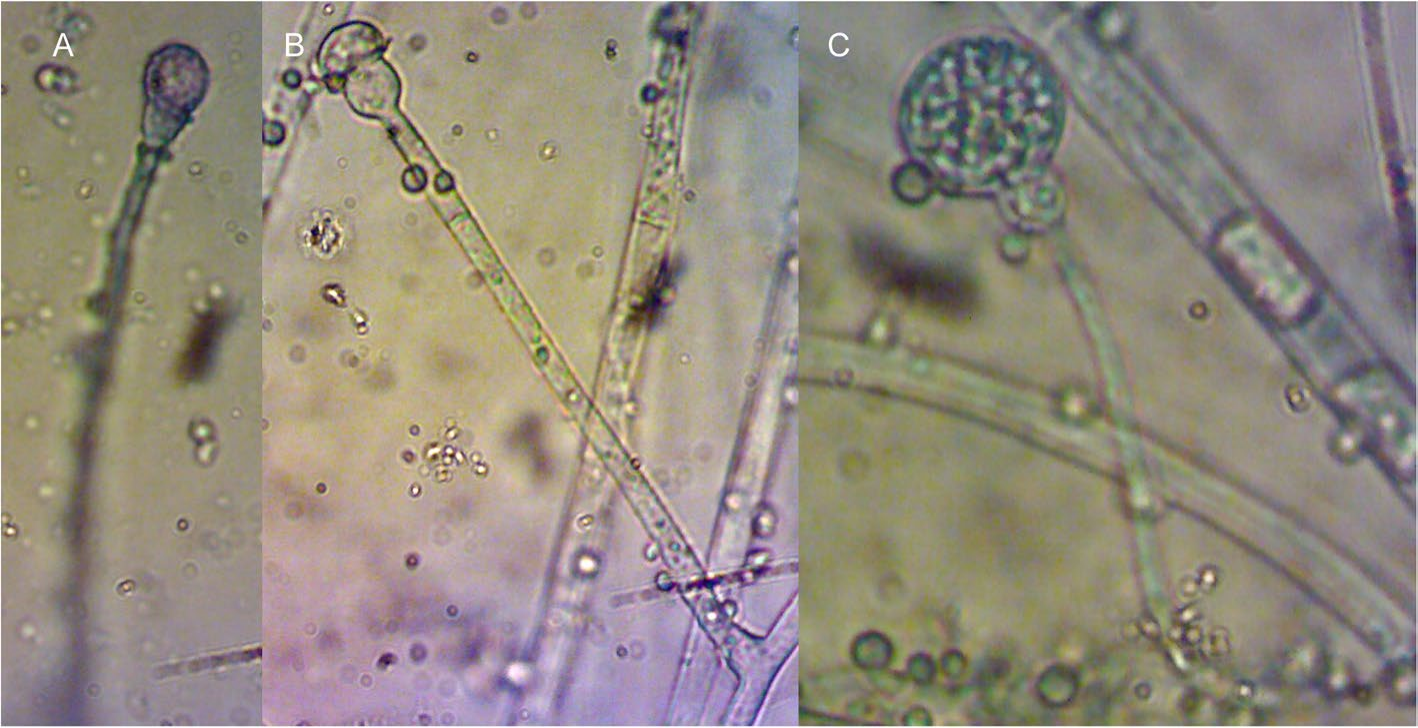
Sporangial characteristics of *G. butleri*. **A** and **B** Developmental stages. **C** Mature stage

Fungus was isolated from the composted material, and pure cultures were developed by picking fungal tips. Mycelial features on PDA medium and microscopic analysis have established the identity of the fungus (Fig. 1). The identity was cross-confirmed by studying the sporangial features and established as *G. butleri* (Fig. 2). The fungus was slow growing and formed a white fluffy and dome-shaped growth on PDA medium. Mycelium was branched with globose sporangia and the sporangiospores oval to flattened on one side.

### Production of degradative enzymes and in vivo bio-solid waste degradation

Compared to *B. subtilis*, the GbKAU strain of *G. butleri* has shown significantly higher levels of laccase and amylase production. Cellulase and glucanase production was higher in *B. subtilis* (Table 1).

**Table 1:** Production of cellulase, laccase, and amylase by *G. butleri* and *B. subtilis*

Fungus	Cellulase (mg of glucose/ ml)		Laccase (U/ml)	Amylase (mg of glucose/ml)
	C_**1**_ + C_**X**_	Glucanase		
*B. subtilis*	0.923	0.530	0.307	0.147
*G. butleri*	0.317	0.427	**0.683**	**0.237**
CD (0.05)	0.045	0.041	0.251	0.017

**Fig. 3:**
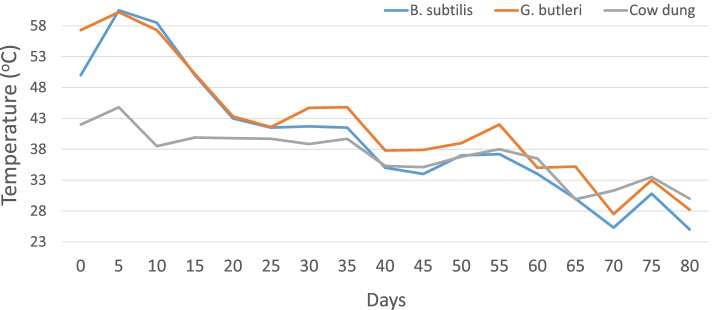
Variation in temperature among different composting treatments during the first 80 days

When *G. butleri*, *B. subtilis*, and cow dung were compared for their solid waste decomposing potential for 80 days, the percent volume reduction was significantly higher on microbial inoculants, compared to cow dung (Table 2). Significantly, higher reduction in the compost volume by *G. butleri* compared to the cow dung-based compost was seen from 40 days of composting, and after 80 days, both the microbes were equally superior. From the beginning itself, *G. butleri* has maintained a higher compost temperature. On 10th day, the compost temperature was higher by 20.17 and 19.34 °C in *B. subtilis* and *G. butleri*, respectively, compared to the compost using cow dung (Fig. 3, Table 2).

**Table 2:** Reduction in volume and heat generation under composting using *G. butleri*, *B. subtilis*, and cow dung

Treatments	% reduction in volume					Temperature °C					
	Days after treatment					Days after treatment					
	10	20	40	60	80	0	10	20	40	60	80.00
*B. subtilis*	38.70	45.19	55.36^a^ (48.08)	59.04^a^ (50.20)	62.43^a^ (52.19)	50.00^a^	58.00^a^	43.17	34.50	33.33	25.33
*G. butleri*	29.94	36.44	46.33^b^ (42.89)	53.389^ab^ (46.94)	58.47^a^ (49.88)	56.66^a^	57.17^a^	43.66	37.33	35.00	27.67
Cow dung	29.65	34.46	42.93^b^ (40.93)	46.89^b^ (43.21)	50.28^b^ (45.16)	42.00^b^	37.83^b^	39.00	35.00	36.16	30.00
CD (0.05)	NS	NS	2.39	4.04	3.37	7.14	7.60	NS	NS	NS	NS

### Sequencing and assembly

Purity of the extracted DNA was tested good for sequencing, and the sequencing on Illumina has given 12,294,196 reads of which 11,188,927 were processed. Raw reads analyzed were of high quality, and the genome features of the strain are presented in Table 3. Reads are made available at SRA database under the BioProject PRJNA639031. The twofold de novo assembly approach used SPAdes 3.11.1 followed by confirmation using ABySS 2.2.4 assembly tool. Best k-mer value of 120 has given the final assembly consisting of 44,639 scaffolds with the draft genome size of 29.8 Mb.

**Table 3:** Genome features of the fungus *G. butleri*

Genome	Features	Value
*G. butleri* strain GbKAU	Illumina reads	12,294,196
	Scaffolds	44639
	Maximum scaffold length	44183
	Minimum scaffold length	114
	Genome size	29.8 Mb
	N50	1136
	No. of genes	11428

### Gene prediction and annotation

**Fig. 4:**
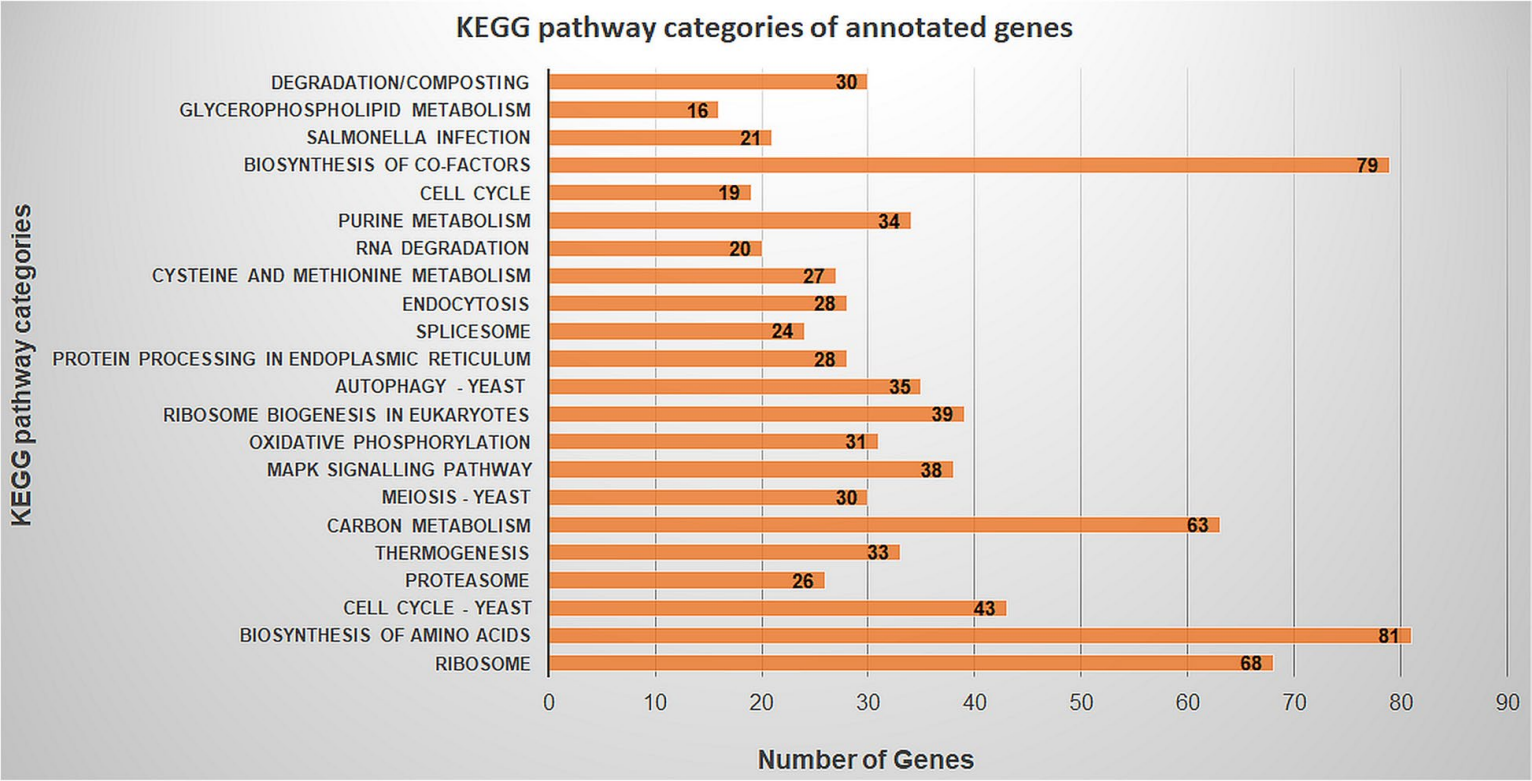
Major KEGG pathway categories of the genes annotated from *G. butleri* genome

Augustus program has predicted 11,428 genes (Table 3), and KAAS annotation has classified the genes into 359 groups involved in diverse pathways (Fig. 4), of which 14 categories represented the enzymes involved in the degradation of macromolecules.

Genes identified from this genome and coding for the enzymes with direct involvement in biodegradation were urea carboxylase, aldehyde dehydrogenase, saccharopine dehydrogenase, dihydrolipoamide dehydrogenase, acetyl-CoA C-acetyltransferase, 2-oxoglutarate dehydrogenase, acyl-CoA dehydrogenase, dihydrolipoamide dehydrogenase, hydroxymethylglutaryl-CoA synthase, 3-hydroxyisobutyryl-CoA hydrolase, 4-aminobutyrate aminotransferase, S-(hydroxymethyl) glutathione dehydrogenase, acyl-CoA oxidase, Delta3-Delta2-enoyl-CoA isomerase, S-(hydroxymethyl)glutathione dehydrogenase, beta-galactosidase, alpha-mannosidase, and hexosaminidase.

**Fig. 5:**
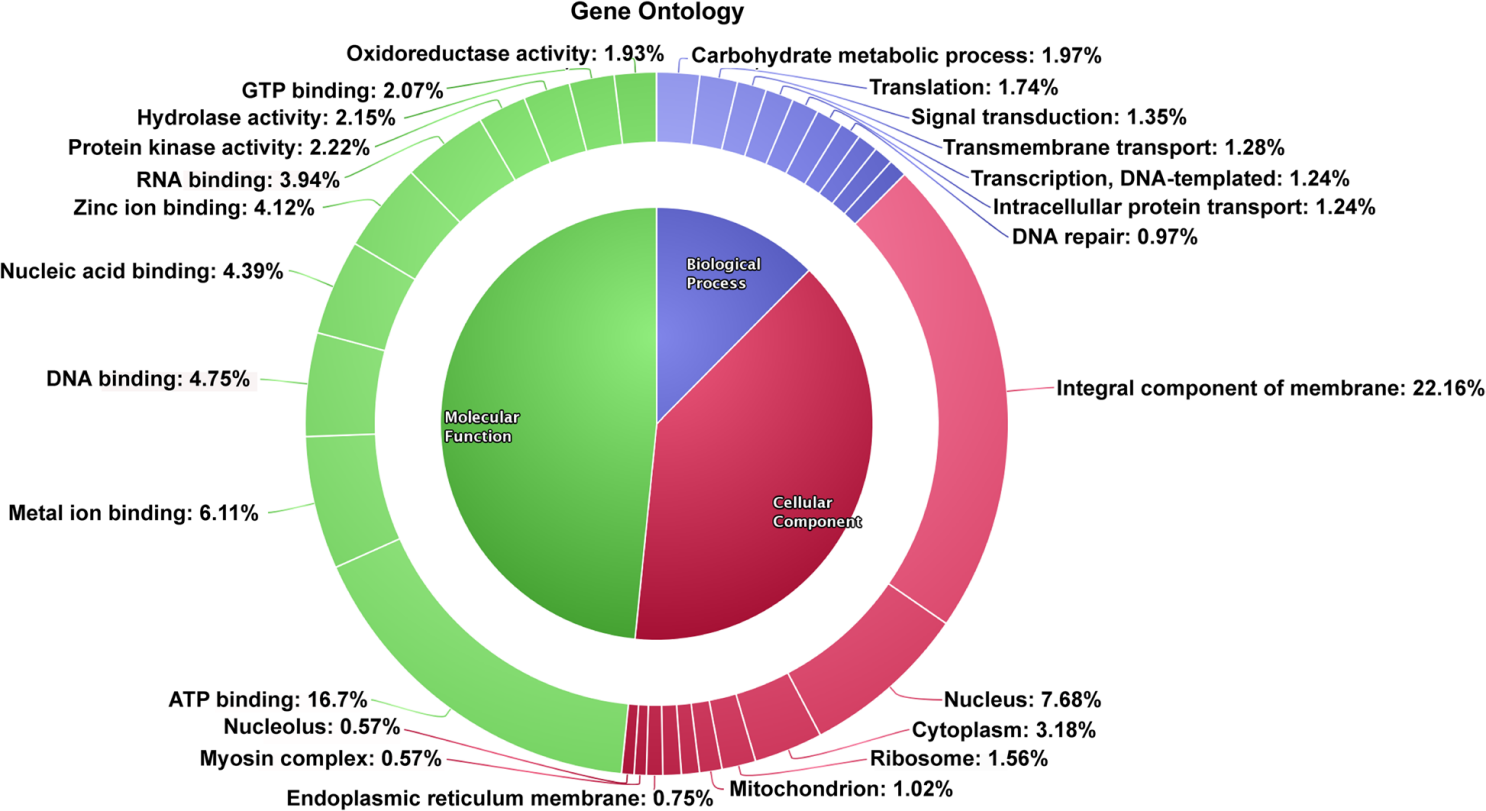
Gene ontology report for *G. butleri* genome

Of the 11,428 genes predicted, 10,024 were annotated by BLASTx-based extensive whole genome annotation (Supplementary material 1). Significant hits were not seen for the remaining genes. Gene Ontology analysis had classified the genes according to the molecular function, biological process, and cellular component. The molecular function part showed that the maximum genes have role in ATP-binding process (16.7 %), while the activities such as oxidoreductase, hydrolase, and protein kinase were performed by 1.93, 2.15, and 2.22 % genes, respectively (Fig. 5). A total of 104,718 SSRs were identified and classified to mono- to hexa-nucleotide repeats. The number of SSRs in each category was mono, 51956; di, 27377; tri, 12965; tetra, 12073; penta, 233; and hexa, 114 (Supplementary material 2). The variant analysis in comparison with *Cunninghamella* genome has revealed 1156 SNPs (Supplementary material 3).

### Variant detection

*Gongronella* and *Cunninghamella* are closely related genera, and very often, distinguishing them is difficult. The finding of 1156 SNPs among these genera by variant analysis is promising for their molecular discrimination by markers targeting the polymorphisms. Similarly, microsatellite markers have extensive use in gene/QTL mapping and tagging. Genome-wide characterization of the microsatellite markers done in this study will be helpful to identify the unique microsatellites in this species, enabling the development of species-specific markers. 

### Comparative genome analysis with other strains

Raw data sequences of seven strains available in SRA database, submitted by the DOE Joint Genome Institute (JGI), were used for the comparative analysis. Similar to our strain, all these strains were sequenced using Illumina, and the layout was paired end. The strains were compared for the number of genes and the presence of already reported biodegradative enzymes. The number of genes in all the strains was comparable to our strain. For example, accession SRX5287802 (*G. butleri* NRRL A-9978 strain) had 11,138 genes, while SRX9171236 (*G. butleri* NRRL5480 strain) had 10,099 genes. While considering the degrading and catabolic enzymes present in those strains, important enzymes found in our strain such as urea carboxylase, aldehyde dehydrogenase, saccharopine dehydrogenase, hydroxymethylglutaryl-CoA synthase, 3-hydroxyisobutyryl-CoA hydrolase, S-(hydroxymethyl) glutathione dehydrogenase, and S-(hydroxymethyl) glutathione dehydrogenase were also found in all the previously reported strains. The presence of these enzymes shall further strengthen the claim about the biodegradation/composting potential of the fungus.

## Discussion

*Gongronella butleri* belonging to the Zygomycetes class of fungal lineage is a widely distributed microbe with industrial and biotechnological applications [9, 15, 18, 19]. Our previous studies have demonstrated the biocomposting potential of this fungus [13]. Western Ghats is a biodiversity hotspot in India, and the microbial consortium isolated from this hotspot, including *G. butleri*, is found to significantly accelerate the degradation of the organic wastes. This microbial consortium is commercialized by the university and is extensively used in the disposal of household organic wastes. Even with a great industrial potential, little is known about its molecular features, and extensive genomic data are missing. Thus, this study aimed to sequence the genome of this important fungus, to annotate the genome, and to identify the genes and enzymes which will have various applications, especially in biodegradation.

### Fungus isolation and morphological confirmation

Establishment of the identity is the most important step while reporting the genome of any fungus. This becomes challenging when the genera associated are genetically and morphologically more similar. Even though the mycelial features on PDA medium and the microscopic analyses were successful to identity of the fungus, cross-verification was done to assure the identity. The mycelial and sporangial characters found in this study were similar to those reported previously [35, 36].

### Degradative enzyme production by the G. butleri strain

The high levels of laccase and amylase is imparting the biodegradation capabilities to this strain. *G. butleri* is already reported to be a good source of amylase [18]. As seen in this work, *B. subtilis* is understood as a good source of cellulase and glucanase enzymes [37]. The levels of enzyme recovery in this study was comparable with those reported in previous studies. This indicated the possibility of exploitation of this fungus in the management of biosolid/municipal solid waste through composting.

### In vivo biosolid waste degradation

Compared to cow dung, *G. butleri* and *B. subtilis* inoculants were superior for their solid waste decomposing potential. The superiority in terms of the reduction in the compost volume and temperature was visible in 40 days. Heat generation is the indirect measure of the respirometric activity during aerobic composting, and hence, this is considered as a measure of the degradative process [38]. These results have clearly shown that *G. butleri* possesses good bio-degradative potential.

### Sequencing and assembly

Although Illumina offers the sequences at short read lengths, the accuracy will be better than other platforms [39]. The twofold de novo assembly approach which uses SPAdes 3.11.1 followed by ABySS 2.2.4 assures an error-free assembly. Several new de novo assemblers, each with unique advantages and disadvantages, have been developed recently for the short reads generated by next-generation sequencing platforms. A dual-fold approach with the tools such as SPAdes and ABySS is reported to give better assemblies [40]. The genome size of 29.8 Mb was comparable with the 33.01 Mb given by the Joint Genome Institute (JGI) (https://mycocosm.jgi.doe.gov/Gonbut1/Gonbut1.info.html) using PacBio (150×) platform that offers a higher read length.

### Gene prediction and annotation

This study has predicted 11,428 genes which are better than the 11,004 genes given by JGI. Bidirectional best hit method followed in this study is identified as the best method for annotating the complete genomes [41].

Annotation had shown that the genome houses many enzymes with direct involvement in biodegradation processes, elucidating the composting potential of this fungus. The enzyme urea carboxylase is identified to be involved in carboxylation in the degradation process [42]. Enzymes aldehyde dehydrogenase is shown to have profound roles in the degradation of organic as well as inorganic complex molecules [43, 44]. During microbial degradation of the polymer PEG, it is initially oxidized to carboxylated PEG by alcohol and aldehyde dehydrogenases and then depolymerized [45]. In aerobic furfural degradation, it is first oxidized to 2-furoic acid by an aldehyde dehydrogenase [46–48]. Enzyme saccharopine dehydrogenase plays pivotal role in lysine degradation by acting on the intermediate saccharopine [49–51]. Similarly, the enzyme dihydrolipoamide dehydrogenase identified from this fungus is known to have important role in the cleavage of the ethoxylate chain of nonionic surfactants [52, 53], whereas 2-oxoglutarate dehydrogenase has roles in L-lysine, L-hydroxylysine, and L-tryptophan degradation pathways [54, 55].

Enzymes hydroxymethylglutaryl-CoA synthase [56] and 3-hydroxyisobutyryl-CoA hydrolase [57, 58] have well-demonstrated catabolic activities. Enzyme 4-aminobutyrate aminotransferase is involved in the degradation of different amino acids [59, 60], whereas the glutathione metabolism-related enzyme identified in this fungus, S-(hydroxymethyl) glutathione dehydrogenase, is involved in microcystin degradation [48, 61]. Acyl-CoA oxidase enzyme is very important for fatty acid degradation in plants [62, 63], and alpha-mannosidase is involved in the degradation of glycoproteins [64, 65]. This array of macromolecule-degrading enzymes explains the composting potential of this strain. Existing reports show that the biodegradation potential of this fungus is such that it degrades even minerals and limestone [66–68]. Recent studies confirm that *G. butleri* is a powerful mineral-solubilizing microorganisms which enhance the nutrient status of the soil, leading to better plant growth and remediation of abandoned mines [69].

## Conclusion

The fungus *G. butleri* belonging to the phylum Zygomycota survives under a wide range of environments and has important biotechnological and industrial applications. The fungus was isolated and purified, and the production of higher levels degradative enzymes laccase and amylase was demonstrated. In vivo evaluation of the fungus for biosolid waste management had shown its efficiency in composting. The fungus was sequenced, and the first draft genome is reported and discussed in this paper. Illumina sequencing at 50× depth had resulted in 29.8 Mb genome harboring 11,428 genes. Of these, many genes encode 14 categories of enzymes associated with the biodegradation process, explaining its composting potential. BLASTx followed by Gene Ontology analysis, SSR mining, and variant analysis had revealed the major molecular features of the genome. Comparative analysis with previously sequenced strains has shown that they have comparable number of genes and similar enzymes. The results have reinforced the composting potential of this fungus.

## Supplementary Information

**Additional file 1: Supplementary material 1.** Annotation for the 10,024 proteins through BLASTx against all fungal protein sequences available at Uniprot Protein Database

**Additional file 2: Supplementary material 2.** Characteristics of the microsatellites mined from *Gongronella butleri* genome

**Additional file 3: Supplementary material 3.** SNPs identified through variant analysis in *Gongronella butleri* genome against *Cunninghamella* genome

## Data Availability

The whole genome data generated in this study is deposited in the NCBI SRA database under the BioProject PRJNA639031.

## Abbreviations

ABySS: Assembly by short sequences
CMC: Chopped meat carbohydrate broth
DNS: 3,5-Dinitrosalicylic acid
EDTA: Ethylenediaminetetraacetic acid
GO: Gene Ontology
KEGG: Kyoto Encyclopedia of Genes and Genomes
PDA: Potato dextrose agar
SDS: Sodium dodecyl sulfate
SRA: Sequence read archive
